# Fibromyalgia is associated with increased 90-day readmission and procedural utilization during readmission after elective primary total hip arthroplasty

**DOI:** 10.1186/s42836-026-00391-w

**Published:** 2026-05-01

**Authors:** David Maman, Yaniv Steinfeld, Yaron Berkovich

**Affiliations:** 1 Technion Israel Institute of Technology, The Ruth and Bruce Rappaport Faculty of Medicine, Technion City, 3200003 Haifa, Israel; 2 Carmel Medical Center, 3436212 Haifa, Israel

**Keywords:** Total hip arthroplasty, Fibromyalgia, Readmission, Procedural utilization, Reoperation, Nationwide Readmissions Database

## Abstract

**Purpose:**

Fibromyalgia is a chronic pain syndrome characterized by central sensitization and frequent comorbidity clustering. Its impact on early readmission and procedural utilization during readmission after elective primary total hip arthroplasty (THA) remains incompletely defined. We evaluated the association between fibromyalgia and 90-day readmission and readmission-associated procedural outcomes after elective primary THA.

**Methods:**

We performed a retrospective cohort study using the Nationwide Readmissions Database (NRD) (2020–2022). Elective primary THA hospitalizations were identified and restricted to procedures performed on hospital day 0. Fibromyalgia status was defined using ICD-10-CM diagnosis code M79.7 (captured in the database as diagnosis codes beginning with M797) during the index hospitalization. To improve comparability, we performed 1:5 propensity score matching without replacement. Outcomes included index hospitalization complications, all-cause 90-day readmission, and readmission-associated procedural outcomes, including component-level revision-type procedures, hip-related reoperation, and any inpatient procedure during readmission.

**Results:**

Among 366,374 elective primary THA hospitalizations, 7,868 patients (2.1%) carried a diagnosis of fibromyalgia. After 1:5 propensity score matching, 46,126 patients remained (7,868 fibromyalgia; 38,258 controls), with standardized mean differences < 0.1 across matched covariates. During the index hospitalization, fibromyalgia was associated with higher rates of blood loss anemia (20.0% vs 16.5%; OR 1.27, 95% CI 1.19–1.35; *p* < 0.001), blood transfusion (3.5% vs 2.8%; OR 1.25, 95% CI 1.09–1.43; *p* = 0.001), pulmonary embolism (0.2% vs 0.1%; OR 2.21, 95% CI 1.20–4.08; *p* = 0.009), sepsis (0.2% vs 0.1%; OR 1.84, 95% CI 1.00–3.41; *p* = 0.048), and urinary tract infection (1.3% vs 1.1%; OR 1.25, 95% CI 1.01–1.55; *p* = 0.042). Ninety-day readmission occurred in 7.4% of patients with fibromyalgia compared with 5.0% of matched controls (OR 1.53, 95% CI 1.39–1.69; *p* < 0.001). During readmission, fibromyalgia was associated with increased odds of component-level revision-type procedures (1.6% vs 1.0%; OR 1.53, 95% CI 1.25–1.87; *p* < 0.001), hip-related reoperation (1.9% vs 1.4%; OR 1.37, 95% CI 1.14–1.65; *p* = 0.001), and any inpatient procedure (4.9% vs 3.4%; OR 1.48, 95% CI 1.32–1.67; *p* < 0.001).

**Conclusions:**

In a nationwide propensity-matched cohort of elective primary THA, fibromyalgia was associated with higher 90-day readmission and greater procedural utilization during readmission. Because important clinical variables and medication exposures are not captured in the NRD, these findings should be interpreted as associative rather than causal.

## Introduction

Total hip arthroplasty (THA) is one of the most successful and cost-effective procedures in modern orthopaedic surgery, with rapidly increasing procedural volumes globally [1, 2]. In the United States alone, annual THA volumes have risen substantially in recent years, reflecting both an aging population and expanding surgical indications [1]. Advances in implant design, surgical technique, and peri-operative protocols, including Enhanced Recovery After Surgery (ERAS) pathways, have contributed to improved short-term outcomes and reduced hospital length of stay [2, 3].

Despite these advances, patient-specific factors remain critical determinants of post-operative outcomes. Fibromyalgia (FM) is a chronic pain disorder characterized by central sensitization, widespread musculoskeletal pain, fatigue, and heightened pain perception [4, 5]. It is increasingly recognized as an important comorbidity in patients undergoing elective orthopaedic procedures [6].

Emerging evidence suggests that fibromyalgia may adversely influence post-operative outcomes following joint arthroplasty. Prior studies have demonstrated that fibromyalgia characteristics independently predict worse pain outcomes and increased opioid consumption after lower-extremity arthroplasty [7]. In total knee arthroplasty, fibromyalgia has been associated with inferior patient-reported outcomes [8].

Similarly, in THA populations, fibromyalgia has been linked to increased post-operative complications and higher healthcare utilization [9, 10]. However, contemporary nationwide data evaluating early readmission and revision-related outcomes in this population remain limited.

Given the increasing volume of elective THA procedures [1] and the growing recognition of centralized pain syndromes as outcome modifiers, a clearer understanding of the impact of fibromyalgia on short-term procedural outcomes is clinically relevant. Improved risk stratification may help refine perioperative counseling and postoperative monitoring strategies for this population. However, because administrative data are vulnerable to residual confounding and coding-based misclassification, nationwide analyses should be interpreted as identifying associations rather than establishing causality.

## Materials and methods

### Data source

We conducted a retrospective cohort study using the Nationwide Readmissions Database (NRD) for the years 2020 through 2022. The NRD is a large, all-payer database designed to support national estimates of readmissions and allows linkage of patients across hospitalizations within a calendar year. All data are de-identified; therefore, this study was considered exempt from institutional review board oversight.

### Cohort identification

Elective primary THA admissions were identified using ICD-10-PCS procedure codes and restricted to hospitalizations in which the procedure occurred on hospital day 0. This restriction was applied to reduce heterogeneity related to delayed operative care, prolonged pre-operative hospitalization, or intercurrent inpatient events before surgery, and to better reflect planned elective arthroplasty pathways. Additional exclusions were applied to focus the cohort on elective cases and to minimize confounding from trauma or emergent indications, consistent with contemporary NRD arthroplasty methodology. To further improve cohort homogeneity, the analysis was restricted to patients undergoing THA for primary osteoarthritis of the hip, identified using ICD-10-CM diagnosis codes beginning with M16 during the index hospitalization. This approach was used to limit heterogeneity related to alternative indications such as osteonecrosis, inflammatory arthritis, dysplasia, or post-traumatic arthritis. For 90-day analyses, index procedures were restricted to those performed early enough within the calendar year to permit complete 90-day follow-up.

### Exposure definition

Fibromyalgia status was defined using ICD-10-CM diagnosis code M79.7, identified in the database as diagnosis codes beginning with M797, recorded during the index hospitalization. Patients were classified as fibromyalgia-positive if at least one qualifying fibromyalgia diagnosis code was present in any diagnosis field during the index admission (Table 1).

**Table 1:** Baseline characteristics of elective primary THA patients by fibromyalgia status (unmatched cohort)

Variable	No Fibromyalgia (*n* = 358,507)	Fibromyalgia (*n* = 7,868)	*p*-value
Age (years), mean ± SD	67.08 ± 10.83	65.72 ± 9.86	< 0.001
Female sex (%)	55.5%	94.3%	< 0.001
Hypertension (%)	52.2%	53.8%	0.004
Dyslipidemia (%)	47.8%	50.2%	< 0.001
Obstructive sleep apnea (%)	12.3%	20.1%	< 0.001
Chronic anemia (%)	5.3%	7.8%	< 0.001
Osteoporosis (%)	4.8%	11.2%	< 0.001
Mental disorders (%)	31.3%	62.8%	< 0.001
Type 2 diabetes (%)	16.5%	20.1%	< 0.001
Chronic kidney disease (%)	8.5%	10.1%	< 0.001
Congestive heart failure (%)	1.4%	1.7%	0.009
Chronic lung disease (%)	6.7%	12.8%	< 0.001
Peripheral vascular disease (%)	1.7%	3.8%	< 0.001
Prior cerebrovascular accident (%)	4.4%	7.0%	< 0.001
Peptic ulcer disease (%)	0.3%	0.4%	0.005
Thyroid disorders (%)	15.8%	29.3%	< 0.001
Obesity (any) (%)	27.4%	36.5%	< 0.001
Robotic surgery (%)	4.4%	4.9%	0.053
Parkinson’s disease (%)	0.6%	0.5%	0.774
Alzheimer’s disease (%)	0.2%	0.3%	0.584
History of MI (%)	3.7%	3.4%	0.110

### Outcomes

Outcomes were evaluated at two levels:

1. Index hospitalization outcomes: peri-operative medical and surgical complications recorded during the index admission, including blood loss anemia, blood transfusion, pulmonary embolism, sepsis, urinary tract infection, and additional index complications as reported in Table 2.
2. Ninety-day outcomes: all-cause 90-day readmission and readmission-associated procedural outcomes, including (a) component-level revision-type procedures during readmission, (b) hip-related reoperation during readmission, and (c) any inpatient procedure code present during readmission. Hip-related reoperation was defined using ICD-10-PCS procedure codes beginning with 0SR, 0SP, or 0S9 in any procedure field during readmission. Component-level revision-type procedures were defined more specifically as ICD-10-PCS codes beginning with 0SP (removal) or selected 0SR subcodes (0SRR, 0SRC, 0SRD), representing removal or replacement of hip prosthetic components during readmission. Any inpatient procedure was defined as the presence of any ICD-10-PCS procedure code during readmission, regardless of anatomic relation. Because ICD-10-PCS coding does not always distinguish with perfect clinical granularity between complete component revision, modular exchange, or other component-related procedures, this endpoint should be interpreted as a revision-type procedural intervention rather than registry-validated implant failure. Readmission diagnoses were summarized using diagnostic grouping and standardized per 10,000 procedures to facilitate clinical interpretability and between-group comparison.

**Table 2:** Index hospitalization complications after 1:5 propensity score matching

Complication	No Fibromyalgia (*n* = 38,258)	Fibromyalgia (*n* = 7,868)	Odds Ratio (95% CI)	*p*-value
Blood loss anaemia	16.5%	20.0%	1.27 (1.19–1.35)	< 0.001
Blood transfusion	2.8%	3.5%	1.25 (1.09–1.43)	0.001
Pulmonary embolism	0.1%	0.2%	2.21 (1.20–4.08)	0.009
Sepsis	0.1%	0.2%	1.84 (1.00–3.41)	0.048
Urinary tract infection	1.1%	1.3%	1.25 (1.01–1.55)	0.042
Post-operative pain	1.7%	2.0%	1.18 (0.99–1.41)	0.063
Dislocation	0.3%	0.3%	1.25 (0.81–1.95)	0.313
Intraoperative fracture	1.0%	1.2%	1.21 (0.96–1.51)	0.108

### Covariates

Baseline covariates included demographic characteristics (including age and sex), and a broad set of comorbidities relevant to elective arthroplasty risk modeling, including but not limited to hypertension, dyslipidemia, obstructive sleep apnea, chronic anemia, osteoporosis, mental health disorders, type 2 diabetes mellitus, chronic kidney disease, congestive heart failure, chronic lung disease, peripheral vascular disease, prior cerebrovascular accident, peptic ulcer disease, severe obesity, thyroid disorders, and obesity, as well as robotic-assisted surgery status when applicable.

### Statistical analysis

Baseline characteristics were compared between fibromyalgia and non-fibromyalgia patients in the unmatched cohort using appropriate univariate tests. Because substantial baseline differences were present, 1:5 propensity score matching without replacement was performed using nearest-neighbor methodology to improve comparability between fibromyalgia and non-fibromyalgia patients. The propensity model incorporated demographic characteristics, hospital-level factors, payer status, comorbidity burden, and procedural variables. Covariates included age (continuous), sex, calendar year of surgery, primary payer status, hospital characteristics (bed size, teaching status, urban versus rural location), and relevant comorbidities, including hypertension, dyslipidemia, obstructive sleep apnea, chronic anemia, osteoporosis, mental health disorders, type 2 diabetes mellitus, chronic kidney disease, congestive heart failure, chronic lung disease, peripheral vascular disease, prior cerebrovascular accident, prior myocardial infarction, peptic ulcer disease, severe obesity, obesity (any), thyroid disorders, Parkinson disease, Alzheimer disease, and robotic-assisted surgery utilization. Post-matching balance was assessed using standardized mean differences, with a threshold of < 0.1 indicating adequate covariate balance. All matched covariates met this criterion. The NRD does not capture anesthesia type, outpatient medication exposure (including opioids, antidepressants, and neuropathic pain medications), implant design, surgical approach, laboratory values, frailty measures, functional status, or detailed psychosocial variables.

## Results

### Baseline characteristics

Among 366,374 elective primary THA hospitalizations, 7,868 patients (2.1%) carried a diagnosis of fibromyalgia. Patients with fibromyalgia were younger than those without fibromyalgia (65.72 ± 9.86 vs 67.08 ± 10.83 years; mean difference 1.36 years, 95% CI 1.12–1.60; *p* < 0.001).

Fibromyalgia patients were overwhelmingly female (94.3% vs 55.5%, *p* < 0.001). They demonstrated a significantly higher burden of comorbidities, including obstructive sleep apnea (20.1% vs 12.3%), chronic anemia(7.8% vs 5.3%), osteoporosis (11.2% vs 4.8%), mental health disorders (62.8% vs 31.3%), type 2 diabetes (20.1% vs 16.5%), chronic kidney disease (10.1% vs 8.5%), chronic lung disease (12.8% vs 6.7%), peripheral vascular disease (3.8% vs 1.7%), prior cerebrovascular accident (7.0% vs 4.4%), and thyroid disorders (29.3% vs 15.8%) (all *p* < 0.001).

No significant differences were observed in Parkinson’s disease, Alzheimer’s disease, or a history of myocardial infarction. Robotic-assisted surgery utilization was similar between groups (4.9% vs 4.4%, *p* = 0.053). As shown in Table 1.

Given these substantial baseline differences, propensity score matching was performed to improve cohort comparability.

### Post-matching cohort

After 1:5 propensity score matching without replacement, 46,126 patients remained in the analytic cohort, including 7,868 patients with fibromyalgia and 38,258 matched controls without fibromyalgia. Post-matching balance diagnostics demonstrated standardized mean differences < 0.1 across all matched covariates, confirming adequate covariate balance between groups.

### Index hospitalization complications

Despite adequate covariate balance following propensity matching, differences in perioperative morbidity during the index hospitalization persisted between cohorts. Patients with fibromyalgia experienced higher rates of blood loss anemia compared with matched controls (20.0% vs 16.5%; OR 1.27, 95% CI 1.19–1.35; *p* < 0.001), as well as higher utilization of blood transfusion (3.5% vs 2.8%; OR 1.25, 95% CI 1.09–1.43; *p* = 0.001). Thromboembolic events were also more frequent in the fibromyalgia cohort, including pulmonary embolism (0.2% vs 0.1%; OR 2.21, 95% CI 1.20–4.08; *p* = 0.009). In addition, fibromyalgia was associated with higher rates of sepsis (0.2% vs 0.1%; OR 1.84, 95% CI 1.00–3.41; *p* = 0.048) and urinary tract infection (1.3% vs 1.1%; OR 1.25, 95% CI 1.01–1.55; *p* = 0.042).

A nonsignificant trend toward higher coding of post-operative pain was observed among patients with fibromyalgia (2.0% vs 1.7%; *p* = 0.063). In contrast, no statistically significant differences were observed in rates of hip dislocation, intra-operative fracture, myocardial infarction, pneumonia, prolonged ventilation, or ileus between groups.

### Ninety-day readmission and readmission-associated procedural outcomes after 1:5 propensity score matching

After 1:5 propensity score matching, fibromyalgia remained independently associated with higher 90-day readmission and greater readmission-associated procedural utilization. Ninety-day readmission occurred in 7.4% of patients with fibromyalgia compared with 5.0% of matched controls (OR 1.53, 95% CI 1.39–1.69; *p* < 0.001). During readmission, fibromyalgia was associated with increased odds of component-level revision-type procedures (1.6% vs 1.0%; OR 1.53, 95% CI 1.25–1.87; *p* < 0.001), hip-related reoperation (1.9% vs 1.4%; OR 1.37, 95% CI 1.14–1.65; *p* = 0.001), and any inpatient procedural intervention (4.9% vs 3.4%; OR 1.48, 95% CI 1.32–1.67; *p* < 0.001) (Table 3).

**Table 3:** Ninety-day readmission and readmission-associated procedural outcomes after 1:5 propensity score matching

Outcome during 90-day follow-up	No Fibromyalgia (*n* = 38,258)	Fibromyalgia (*n* = 7,868)	Odds Ratio (95% CI)	*p*-value
Any 90-day readmission	5.0%	7.4%	1.533 (1.393–1.687)	< 0.001
Component-level revision-type procedure during readmission	1.0%	1.6%	1.526 (1.245–1.871)	< 0.001
Hip-related reoperation during readmission	1.4%	1.9%	1.367 (1.136–1.645)	0.001
Any procedure during readmission	3.4%	4.9%	1.483 (1.321–1.666)	< 0.001

### Ninety-day readmission diagnoses per 10,000 primary THA procedures after 1:5 propensity score matching

When standardized to 10,000 procedures, fibromyalgia was associated with higher rates of mechanical prosthesis complications (60 vs 40), and sepsis/bacteremia (40 vs 30), with additional increases in acute kidney injury and cardiopulmonary complications. The overall distribution of readmission diagnoses differed significantly between groups (χ^2^ = 44.1, *p* = 0.001) (Table 4).

**Table 4:** Ninety-day readmission diagnoses per 10,000 primary THA procedures after 1:5 propensity score matching

Readmission Diagnosis	Non-Fibromyalgia (per 10,000)	Fibromyalgia (per 10,000)
Mechanical prosthesis issue	40	60
Sepsis/bacteremia	30	40
Acute kidney injury	10	20
Respiratory infection/failure	10	20
Heart failure/Arrhythmia	10	20
Post-operative pain/hematoma	20	20
PJI/post-operative infection	30	30
Wound dehiscence	10	10
Cellulitis/SSTI	10	10
Pulmonary embolism (PE)	10	10

*Abbreviation: PJI, peri-prosthetic joint infection; SSTI, skin and soft tissue infection; PE, pulmonary embolism. Diagnosis categories represent grouped ICD-10-CM readmission diagnoses summarized into clinically interpretable categories for comparative reporting*

## Discussion

In this national propensity-matched analysis of elective primary THA performed for osteoarthritis, fibromyalgia was associated with higher 90-day readmission and greater readmission-associated procedural utilization. These associations persisted despite good balance across measured covariates after matching; however, they should be interpreted cautiously given the potential for residual confounding and the inherent limitations of administrative data. Rather than establishing a direct causal effect of fibromyalgia itself, the present findings suggest that fibromyalgia may function as a marker of increased early post-operative utilization and peri-operative vulnerability in elective THA populations [11–13]. In addition, the length of stay was not analyzed in the present study. Differences in hospitalization duration between groups could influence the opportunity for complication detection and coding during the index admission and should be considered when interpreting these findings. These findings should therefore be interpreted within the framework of observational data, where associations may reflect both underlying patient vulnerability and unmeasured care pathway differences rather than direct causal effects.

An important consideration is the potential for residual confounding related to variables not captured in the NRD. Patients with fibromyalgia may differ systematically from controls with respect to chronic opioid use, antidepressant exposure, gabapentinoid therapy, sleep disturbance, pain catastrophizing, mental health burden, and broader psychosocial vulnerability. These factors may plausibly influence post-operative pain, healthcare utilization, complication coding, readmission risk, and the likelihood of procedural intervention during readmission. In addition, anesthesia technique may influence early post-operative outcomes after THA, but this variable is not available in the NRD and therefore could not be assessed or adjusted for in the present study. Accordingly, the present findings should not be interpreted as evidence of a direct causal effect of fibromyalgia itself.

### Interpretation of 90-day readmission and readmission-associated procedural outcomes

The magnitude of the readmission signal in the matched cohort is notable: 90-day readmission occurred in 7.4% of fibromyalgia patients versus 5.0% of matched controls, with higher odds of component-level revision-type procedures, hip-related reoperation, and any procedure during readmission. This pattern suggests that fibromyalgia may be associated not only with higher readmission frequency but also with a greater likelihood of procedural care during readmission. However, because these outcomes were defined using administrative procedural coding, they should be interpreted as readmission-associated procedural utilization rather than as definitive evidence of registry-validated implant failure.

Although the observed differences were statistically significant, their absolute magnitude should also be considered. For example, 90-day readmission differed by 2.4 percentage points (7.4% vs 5.0%), and component-level revision-type procedures differed by 0.6 percentage points (1.6% vs 1.0%). These differences remain clinically relevant at a population level, particularly in a high-volume procedure such as THA, but they should not be interpreted as indicating large absolute risk increases for individual patients.

The definition of component-level revision-type procedures in the present study was based on ICD-10-PCS coding for removal and selected replacement procedures involving hip prosthetic components. These codes capture clinically meaningful surgical interventions but may include a spectrum of procedures ranging from component removal or modular exchange to more extensive revision procedures. This coding-based definition may help explain why revision-type procedural rates differed between groups, even though specific readmission diagnoses classically associated with early revision, such as dislocation, peri-prosthetic fracture, or infection, were not individually elevated.

Similarly, the difference between hip-related reoperation rates and rates of any inpatient procedure during readmission indicates that a substantial proportion of readmission procedures were not directly hip-focused. These likely reflect broader inpatient procedural care delivered during readmission rather than arthroplasty-specific surgical failure alone, and this distinction is important when interpreting the clinical significance of the utilization findings.

Several mechanisms may contribute to the observed associations. Fibromyalgia overlaps conceptually with central sensitization and nociplastic pain mechanisms, and central sensitization syndromes have been associated with worse outcomes after THA in contemporary datasets [12]. Pain sensitization features are also prevalent in hip osteoarthritis populations pre-operatively, suggesting that some THA candidates may enter surgery with altered pain processing and reduced tolerance for peri-operative stressors [13]. These factors may contribute to increased early post-operative distress, reduced rehabilitation engagement, and a lower threshold for acute-care presentation.

At the same time, unmeasured psychosocial and behavioral variables likely contribute to residual confounding and may also mediate risk. Pain catastrophizing, depression, anxiety, sleep disturbance, and chronic opioid exposure have all been associated with worse arthroplasty outcomes [14–20]. Because these factors are not captured with sufficient granularity in the NRD, fibromyalgia in this context may partly function as a proxy for broader vulnerability domains that are incompletely measured in administrative datasets.

### Index hospitalization morbidity: signal beyond pain

We observed higher rates of blood loss anemia, transfusion, pulmonary embolism, sepsis, and urinary tract infection among fibromyalgia patients during the index hospitalization after propensity matching. However, several of these outcomes were rare, and some differences were modest in absolute terms. Accordingly, these findings should be interpreted cautiously and viewed as supportive signals rather than definitive evidence of materially increased risk across all perioperative complications. While administrative data cannot determine whether this reflects differences in physiologic reserve, mobilization patterns, perioperative medications, or care pathways, contemporary literature increasingly emphasizes that factors such as sleep quality, mood state, and frailty influence recovery and complications after THA [15–19]. Notably, validated frailty constructs (including simplified frailty indices) have shown predictive value for adverse outcomes and resource utilization in hip arthroplasty populations, and fibromyalgia may partially proxy for vulnerability domains that those tools attempt to quantify more directly (e.g., functional limitation, resilience, and physiologic reserve) [18, 19].

### Specificity of risk pattern and mechanical endpoints

We did not observe uniform elevation across all mechanical or respiratory endpoints (e.g., dislocation, intra-operative fracture, prolonged ventilation, ileus), which provides specificity: risk elevation appears concentrated in early medical complications and readmission-associated escalation rather than across every adverse event category. This distinction is clinically important. For example, if dislocation risk is not globally increased, surgeons may reasonably focus mitigation strategies on medical optimization, early mobilization support, and structured follow-up rather than presuming a broadly heightened mechanical failure profile. At the same time, when mechanical concerns do arise, contemporary implant and approach considerations (e.g., dual mobility constructs) may reduce dislocation risk across approaches, potentially buffering one pathway of readmission in selected patients [11]. This is not to imply dual mobility is indicated for fibromyalgia per se, but rather that mechanical risk-mitigation tools exist and should be applied based on standard indications and individualized instability risk.

### Readmission diagnosis distribution: broader drivers of utilization

When readmission diagnoses were standardized per 10,000 procedures, fibromyalgia patients demonstrated higher rates of gastrointestinal complications and mechanical prosthesis issues, along with increases in sepsis and select cardiopulmonary and renal categories. Although diagnosis groupings simplify heterogeneous ICD-10 codes, the clinically interpretable message is that fibromyalgia patients experience a broader spectrum of readmission drivers spanning both medical and surgery-related pathways. This aligns with the concept that pain sensitization and associated comorbidity clustering can shape recovery across multiple organ-system domains, not solely pain severity [12, 13, 15, 17].

### Clinical implications

These findings have practical implications for pre-operative counseling and peri-operative planning. Fibromyalgia is easily identifiable at baseline and may help flag patients who could benefit from enhanced recovery resources and closer early outpatient monitoring. In addition to standard medical optimization, targeted strategies that address modifiable vulnerability domains may be relevant: structured peri-operative pain plans, sleep optimization, and proactive management of depression/anxiety may improve recovery trajectories and reduce downstream utilization in susceptible patients [15–17]. The broader literature also supports psychosocial risk modification (e.g., catastrophizing-focused interventions) as feasible and potentially impactful in arthroplasty-related recovery, suggesting a pathway for pragmatic perioperative programs that extend beyond purely biomedical risk correction [14].

Importantly, the higher rates of readmission-associated procedural outcomes suggest that post-operative concerns in patients with fibromyalgia should be evaluated carefully rather than assumed to reflect pain amplification alone. A structured and cautious approach to post-operative assessment may be particularly valuable when these patients present early after surgery, as some may require inpatient procedural management during readmission.

From a systems perspective, incorporating fibromyalgia into THA risk stratification may improve the prediction of early utilization and adverse events. Because frailty indices and psychosocial measures both demonstrate predictive value in arthroplasty outcomes research, future models may perform best when they combine traditional medical comorbidities with vulnerability constructs (frailty, sleep, mental health, opioid exposure) that are often incompletely captured in administrative datasets [18–20]. Given the predominance of female patients in the fibromyalgia cohort, these findings primarily reflect outcomes in women and may not be fully generalizable to male patients.

## Limitations

This study has limitations inherent to administrative database research. First, exposures and outcomes are defined by ICD-10 coding, introducing potential misclassification. Fibromyalgia coding may under-capture clinically meaningful disease, and coding intensity may vary across hospitals and patient subgroups. Procedural outcomes were also defined using ICD-10-PCS coding and may include a range of component-related interventions. These codes do not always allow precise differentiation between major full-component revision and more limited procedures such as component removal or modular exchange. Second, the NRD lacks granular clinical detail, including anesthesia type, implant design, surgical approach, operative time, intra-operative blood loss, laboratory values, pain severity, functional status, frailty measures, baseline opioid exposure, outpatient medication use, social support, discharge disposition, outpatient complications, ERAS-related care pathways, VTE prophylaxis strategies, and patient-reported outcomes, limiting mechanistic inference and the ability to fully adjust for psychosocial and vulnerability domains that plausibly drive risk [14, 18–20]. Third, although propensity matching achieved excellent balance across measured covariates, residual confounding from unmeasured factors remains possible. Fourth, although the cohort was restricted to elective primary THA performed for osteoarthritis, residual heterogeneity in disease severity, deformity, and pre-operative functional status could not be assessed due to database limitations. Fifth, linkage is limited to within-year readmissions, which is why analyses were restricted to ensure a complete 90-day follow-up. Finally, because the fibromyalgia cohort remained overwhelmingly female even after matching, the findings may not be fully generalizable to male patients with fibromyalgia. In addition, because the NRD reflects U.S. inpatient practice, these findings may not fully generalize to international populations, outpatient or same-day THA pathways, or centers using substantially different peri-operative protocols.

Despite these limitations, the NRD provides robust national estimates and longitudinal readmission capture. The consistency of findings across readmission and readmission-associated procedural outcomes suggests that fibromyalgia may identify patients at increased risk of early post-operative utilization following elective primary THA. However, given the limitations of administrative data and the potential for residual confounding, these findings should be interpreted cautiously and as associative rather than causal [12–15, 17–20].

## Conclusion

In a nationwide propensity-matched cohort of elective primary THA patients, fibromyalgia was associated with higher 90-day readmission and greater readmission-associated procedural utilization. Fibromyalgia was also associated with selected index hospitalization complications after matching. Because important clinical variables and medication exposures are not captured in the NRD, these findings should be interpreted as associative rather than causal. Nevertheless, fibromyalgia may represent a clinically useful marker of increased early post-operative utilization following elective primary THA. Future studies incorporating granular clinical, psychosocial, and medication-level data are warranted to better define underlying mechanisms and potential targets for intervention.

## Data Availability

This study used publicly available data from the HCUP Nationwide Readmissions Database (NRD). Data can be obtained directly from HCUP (https://hcup-us.ahrq.gov/db/nation/nis/nisdbdocumentation.jsp) upon purchase.
